# Exosome-based strategies for periodontal regeneration: Current insights and future prospects (Review)

**DOI:** 10.3892/mi.2026.334

**Published:** 2026-08-07

**Authors:** Gayathri Kannan, Gayathri Kumar, Potluri Leela Ravishankar, Gracelin Solomon, Kalaivani Varatharajan, Viola Esther Paneerselvan

**Affiliations:** Department of Periodontology, SRM Kattankulathur Dental College and Hospital, Faculty of Medicine and Health Sciences, SRM Institute of Science and Technology, Chengalpattu, Tamil Nadu 603203, India

**Keywords:** exosomes, periodontal diseases, periodontal regeneration, mesenchymal stem cells, bone regeneration

## Abstract

The tooth-supporting structures gradually deteriorate due to periodontal disease and standard regenerative therapies are currently being modified using adjunctive strategies to optimise the treatment outcomes. Nanosized extracellular vesicles called exosomes, which contain bioactive molecules, function as mediators of immunomodulation, regeneration, and tissue repair. Recent research indicates that exosomes may enhance periodontal regeneration through immunomodulation, osteogenesis and angiogenesis from various stem cells. It is suggested that exosomes promote fibroblast proliferation, reduce inflammation and accelerate bone/cementum regeneration. Scaffold-loaded exosomes provide sustained release. The purpose of the present review was to discuss the functions of exosomes and their potential to be used as therapeutic agents in periodontal regeneration. For this purpose, the terms ‘Exosomes’, ‘Extracellular Vesicles’ and ‘Periodontal Regeneration’ were searched in Google Scholar, Web of Science and PubMed databases to identify articles in the English language published up to June, 2025. Grey literature was searched through an internet-based free tool created by the Canadian Agency for Drugs and Technologies in Health. Two independent reviewers evaluated the titles and abstracts of the retrieved studies, followed by a detailed review of the full texts to extract information relevant to the research objective. While further clinical research is required to validate their regenerative potential, the evidence generally points to exosomes as promising therapeutic agents. Although the available data are encouraging, standardized procedures and clinical trials are required before they can be routinely used in clinical settings, to address challenges with delivery and isolation.

## 1. Introduction

Chronic periodontitis, as one of the most prevalent bacterial infectious oral diseases, causes gingival inflammation, the loss of periodontal attachment, destruction of alveolar bone, and ultimately, tooth loss (1). While stratifying the 2009-2010 National Health and Nutrition Examination Survey (NHANES) data by patient age, the prevalence of periodontal disease increases from 24.4% in individuals aged 30-34 years to 70.1% in those aged ≥65 years. Atherosclerosis and Alzheimer's disease are some of the age-related conditions that have been connected to periodontitis and have more severe mortality and morbidity profiles (2). (As *Porphyromonas gingivalis* (*P. gingivalis*) has a significant impact on the local microbiota, it is regarded as a ‘keystone’ pathogen in periodontal disease. *P. gingivalis* disrupts innate immunity, causing dysbiosis, in which symbiotic commensals turn into accessory pathogens via unknown methods (2). Although current periodontal therapy is targeted at the reduction or elimination of disease progression, the regeneration of the lost periodontal structures is however, unsuccessful or compromised (3). During the past decades, regenerative periodontal therapy used guided procedures; however, the fact of predictable regenerative outcomes remains questionable (4).

Almost 30 years have passed since the inception of tissue engineering and regenerative medicine. Studies are being carried out to promote predictable and successful regeneration (5); yet, the complexity of the oral environment local inflammatory milieu and the tissue damage brought on by periodontitis may be the limiting factors for successful therapeutic outcomes. To achieve the predictable and successful outcomes, further studies on newer agents that may enhance regeneration and pave way to overcome these challenges are required. Multipotent stem cells, or mesenchymal stem cells (MSCs) can self-renew and develop in multiple ways. MSC-derived extracellular vesicles (EVs) are deemed to be the most efficient substitutes for MSCs due to their low immunogenicity, high safety and variety of bioactivities; as with their parent MSCs, these EVs can exert immunomodulatory effects and stimulate osteogenesis, cementogenesis and angiogenesis (6).

The MSC secretory profile is composed of exosomes that play a pivotal role in MSC cytotherapy. Exosomes are composed of various membrane proteins, lipoproteins, heat shock proteins and transport-related proteins. Recent research has demonstrated that exosomes secreted by MSCs can initiate tissue regeneration and can potentially be used as therapeutic agents for various diseases (7). Specifically, the importance of exosomes in the development and treatment of periodontal disease is addressed in the present review, and recent breakthroughs in exosome therapy and regeneration are also mentioned (1).

## 2. Exosomes

Exosomes are endocytic EVs and can be secreted by various cell types. Three distinct stages are recognizable in exosomal biogenesis: i) Endocytic vesicles are formed by the plasma membrane. Early endosomes are created when endocytic vesicles fuse, and these endosomes eventually develop into late endosomes. ii) Intraluminal vesicles (ILVs) are created when the late endosome membranes budding inward. The term multivesicular bodies (MVBs) refers to the accumulation of ILVs in late endosomes. iii) ILVs, often referred to as exosomes, are released when MVBs fuse with the plasma membrane (8). Exosomes are lipid bilayer nanoparticles 30-150 nm-diameter (9). There are numerous factors outside the cell, including cell type, serum conditions, cytokines and growth hormones, that affect the production of exosomes. Exosomes are heterogeneous based on the particular shape, content and function (10). Exosomes are also membrane-bound carriers. They are composed of metabolites, proteins and nucleic acids that reflect the physiological state and constitution of the donor cell (11). Exosomes tend to be highly proteinaceous; exosomal proteins play various roles, ranging from tetraspanins (CD9, CD63, CD81 and CD82) involved in cell penetration, invasion and fusion processes to heat shock proteins (HSPs; HSP70 and HSP90), which are implicated in antigen presentation and binding as components of the stress response; some proteins implicated in exosome release (ALIX-TSG101) (12). These molecules not only aid in the identification of exosomes, but also enable them to specifically target recipient cells. Exosomes can reach the distant tissues or organs via the circulatory system after being taken up by recipient cells through autocrine or paracrine signalling; thus, they contribute to multiple physiological and pathological processes involved in tissue repair. Previous studies have also revealed that miRNAs carried within exosomes can induce specific biological effects in recipient cells (5,10).

## 3. Functions of exosomes

Exosomes are extracellular vesicles released by cells that facilitate the transfer of proteins and RNAs across cells (13). Through their paracrine action, exosomes can be employed in tissue regeneration therapies, specifically via influencing cellular activities associated with inflammation, healing, matrix remodelling, differentiation and death (14). Three mechanisms can facilitate the transmission of intercellular signals: Direct membrane fusion, receptor-ligand interaction, or endocytosis/internalization followed by fusing with the endosomal limiting membrane of the receiving cells (15). Cell division, apoptosis and proliferation are all directly related to healthy tissue growth and regeneration. Various growth factors, such as epithelial growth factor, vascular endothelial growth factor (VEGF) and fibroblast growth factor (FGF) are present in exosomes and play a role in cell division and proliferation. As demonstrated in the study by Liang *et al* (16), exosomes produced from MSCs activated the AKT/mTOR pathway, which in turn promoted angiogenesis and bone repair. Through the hypoxia-inducible factor 1α pathway, exosomes have been shown to accelerate fracture healing, stimulate angiogenesis and activate VEGF expression (17).

## 4. Exosomes in periodontal tissue repair

Numerous studies have highlighted that, due to their potent repair-promoting properties, MSC derivatives including EVs, exosomes, matrix vesicles and apoptotic bodies can be utilized as substitutes for MSCs in order to support periodontal tissue regeneration. MSC-derived exosomes may improve cell viability and alter gene expression by triggering pro-survival signalling in periodontal ligament cells. The pro-survival AKT and ERK signalling pathways may be activated by adenosine receptors, and related genes may be expressed to promote the migration and proliferation of periodontal ligament cells. MSC exosomes contain CD73/ecto5'-nucleotidase, which hydrolyses extracellular adenosine monophosphate to produce extracellular adenosine (14). When exosomes are released by cells, they function as messengers and are absorbed by recipient cells through membrane fusion, endocytosis/phagocytosis, or binding to target cell receptors. This allows them to transfer different bioactive substances and contributes to intercellular communication (18).

**Figure 1:**
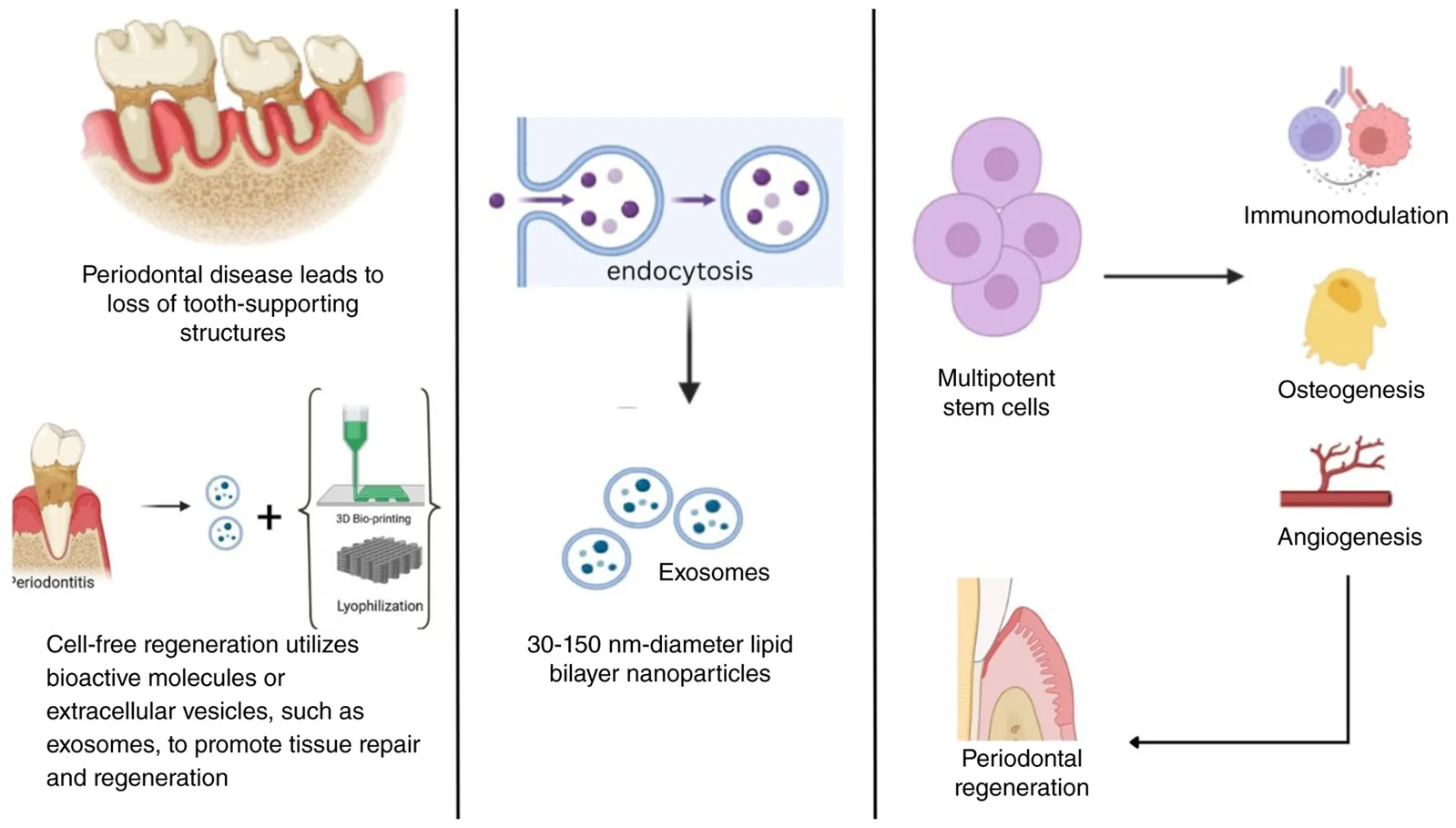
Illustration of the overview of role of exosomes in periodontal regeneration.

**Figure 2:**
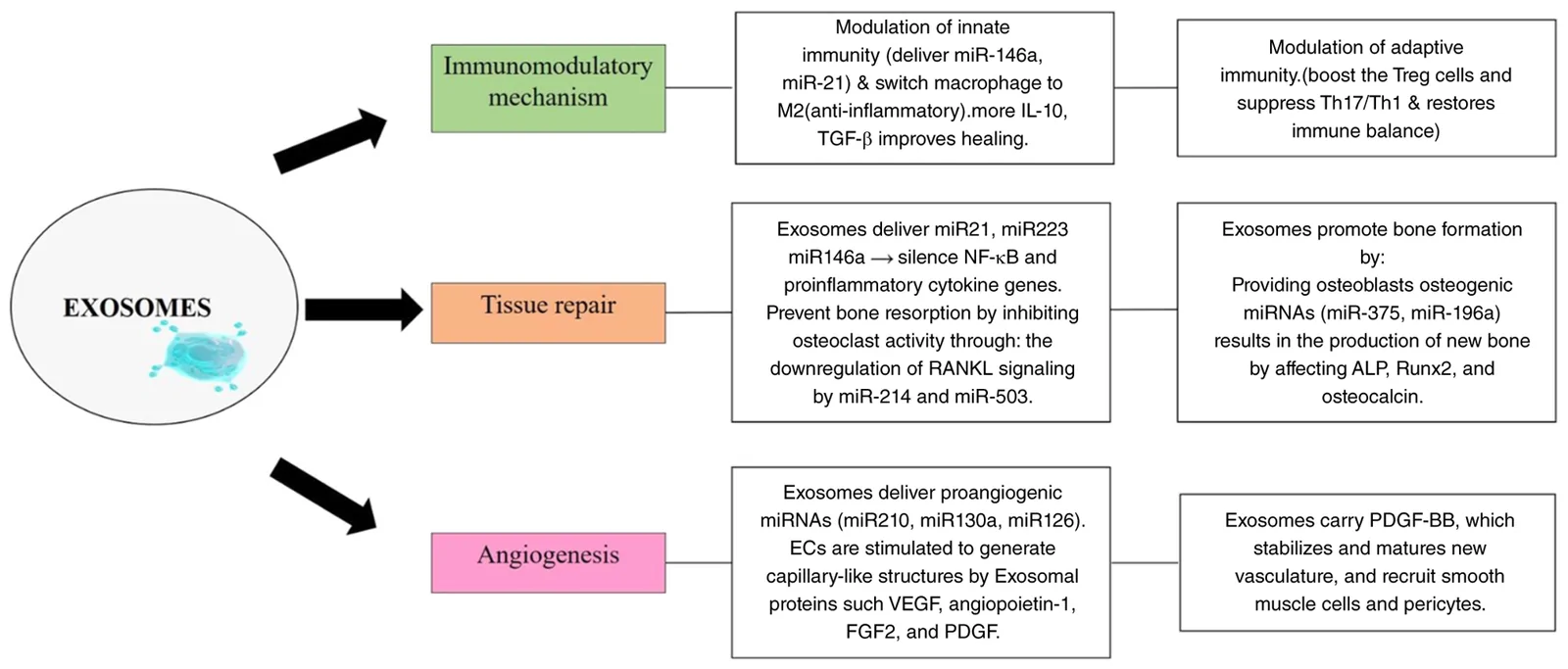
Illustration of the multiple functions of exosomes and their role in supporting periodontal regeneration through three main processes: Angiogenesis, tissue repair and immunomodulation. ECs, endothelial cells; VEGF, vascular endothelial growth factor; FGF2, fibroblast growth factor 2; PDGF, platelet-derived growth factor.

Both innate and adaptive immune responses are regulated by exosomes (11). By promoting macrophage polarization from the M1 to the M2 phenotype, the delivery of miR-146a and miR-21 increases anti-inflammatory activity through the release of TGF-β and IL-10(19). They support tissue repair and immunological homeostasis by increasing Treg cell activity and inhibiting Th17/Th1 responses (13). Exosomal miR-21, miR-223 and miR-146a decrease osteoclast-mediated bone resorption by downregulating RANKL and inhibiting NF-κB signalling and pro-inflammatory cytokine production (20,21). Osteogenic miRNAs, such as miR-196a, increase the production of ALP, Runx2 and osteocalcin in osteoblasts, which promotes the development of new bone (20). Through the VEGF, angiopoietin-1, FGF2 and platelet-derived growth factor (PDGF) pathways, pro-angiogenic miRNAs (miR-210, miR-130a and miR-126) that are delivered by exosomes stimulate endothelial cells to produce capillary-like structures (18,20,22). Additionally, they carry PDGF-BB, which recruits smooth muscle cells and pericytes to sustain vascular integrity by stabilizing and maturing new vasculature (18,23) (Figs. 1 and 2).

Exosomes have been shown to regulate the expression of pro-inflammatory cytokines, such as interleukin (IL)-6 IL-1β and tumour necrosis factor α (TNF-α) by suppressing pathways, such as the Th17/Treg/miR- 155-5p/SIRT1 and IL-6/JAK2/STAT3 pathways (20). Alternatively, M2 macrophages secrete anti- inflammatory cytokines. This alteration provides an environment that supports tissue regeneration (24). Alveolar bone resorption is facilitated by the production of several pro-inflammatory factors (IL1β, TNF-α and IL-6) due to an imbalance favouring M1 macrophages in periodontitis. Additionally, M1 macrophages stimulate the RANKL pathway, which increases bone resorption by stimulating osteoclast differentiation in periodontal tissues. On the other hand, M2 macrophages release anti-inflammatory cytokines (VEGF, TGF-β and IL-10), which support bone healing and immune homeostasis (20). EVs from polarized macrophages may also have an impact on osteogenesis. Kang *et al* (25) found the comparatively high expression of miR-155 in M1 EVs and miR-378a in M2 EVs based on miRNA-sequence analysis of EV cargo. The BMP2 pathway is the specific target of miR-378a, a positive regulator of osteogenesis, and miR-155 is known to play an essential role in inflammatory signalling (25). In order to identify specific molecular mechanisms, Nakao *et al* (19) investigated the therapeutic effect of TNF-α preconditioned- exosomes from gingival tissue-derived MSCs on ligature-induced periodontitis in mice. Exosomal miR-1260b was a crucial regulator for preventing inflammatory bone loss, and TNF-α-induced exosomal CD73 expression helped to polarize M2 macrophages (19). A numb er of previous studies have focused on role of exosomes in periodontal regeneration and these are summarized in Table I (26-32).

## 5. Applications of exosomes in periodontal regeneration

A previous study examined the impact of periodontal ligament stem cell (PDLSC)-derived exosomes (PDLSCs-Exos) on the proliferation and osteogenic differentiation of bone marrow-derived MSCs (BMSCs) using CCK-8 assay and alkaline phosphatase staining (27). Alveolar bone defects were surgically induced mesial to the bilateral maxillary first molars of rats *in vivo*. Bone regeneration was detected by haematoxylin and eosin staining, Masson's trichrome staining and micro-computed tomography. The results indicated that PDLSCs-Exos significantly accelerated BMSC proliferation and osteogenic differentiation. In addition, rats receiving the exosome-hydrogel composite exhibited significantly greater new bone formation at the site of defects when compared to the control and hydrogel groups (27).

The effectiveness of human bone marrow MSC-derived exosomes (HBMSC-Exos) in modulating periodontitis was explored in another study (33). Exosomes were harvested from culture supernatants using a sequence of centrifugations, namely 500 x g for 10 min, 2,000 x g for 10 min and 10,000 x g for 30 min, before being subjected to ultracentrifugation at 100,000 x g for 70 min For the removal of further protein contaminants, pellets were washed and again centrifuged at 100,000 x g for 70 min in 0.9% NaCl, and then resuspended and stored at -80˚C. *In vitro*, HBMSC-Exos inhibited macrophage inflammatory response induced by *P. gingivalis*. *In vivo*, treatment with HBMSC-Exos relieved periodontal inflammation and inhibited alveolar bone loss in experimental periodontitis, indicating their therapeutic potential as a host modulating strategy for periodontitis (33).

Another study also demonstrated that exosomes derived from dental pulp stem cells (DPSC-Exos) proved to have therapeutic potential in regulating immune responses and periodontal repair (34). Periodontitis, which is induced by host immuno-inflammatory responses to infection, results in an imbalance between anti- and pro-inflammatory macrophages. DPSC-Exo-loaded chitosan hydrogel (DPSC-Exo/CS) successfully promoted the repair of alveolar bone and periodontal epithelium in murine models. Gene Ontology term enrichment analysis demonstrated that this effect was achieved through the regulation of immune and inflammatory pathways. miRNA sequencing identified miR-1246 as the most prevalent miRNA in DPSC-Exos, driving macrophage polarization from pro-inflammatory to anti-inflammatory phenotype. The knockdown of miR-1246 using antagomir markedly reduced the therapeutic benefit of DPSC-Exo/CS, validating its pivotal regulatory function in periodontal regeneration (34).

The modification of the surface of exosomes is a vital function in the activity and biological activity, which can compensate for the limitation of the natural exosomes. A critical technological skill for expanding the applications of exosomes is their chemical modification, which may increase the stability of loading, prevent immune detection and target exosomes (35). Furthermore, non-covalent methods such as binding of ligands and receptors, as well as hydrophobic and electrostatic insertion, may be applied for designing, modifying and remodelling the exosome membrane. For the integrity of the exosome membrane, as well as for enhancing their applications in fluorescence, drug delivery and targeting, exosomes modified by exosome engineering techniques are highly efficient and have no adverse effects. Only protein and peptide components synthesized by genetic material may be encapsulated by designed exosomes, and this method has a number of drawbacks (35).

In recent times, drug delivery and regenerative medicine have also exhibited interest in using exosome mimetics, which are prepared using unconventional techniques, such as extrusion. Among various techniques, metal-phenolic network technology was previously found to have immense potential compared to other techniques. As previously demonstrated, tannic acid (TA)-Fe^3+^-coated exosome mimetics led to the increased resistance and improved preservation of particles and proteins (31). EM@[TA-Fe^3+^] also led to a significant reduction in the proliferation of *P. gingivalis*. Pro-inflammatory cytokines, including IL-6, IL-1B, and IL-8, were found to be reduced in BMSCs by EM@[TA-Fe^3+^] (31).

Exosomes, as opposed to other nanocarriers, such as liposomes and polymeric nanoparticles, are naturally occurring carriers for genetic material transfer between cells and mediators of gene expression in recipient cells. They are found in numerous bodily fluids and have several advantages over conventional synthetic delivery vectors, such as direct drug administration to cells, improved biocompatibility, increased stability, less immunogenicity and high blood stability (36).

## 6. Standardization and comparative analysis of exosome isolation methods

It is possible to separate large numbers of EVs from serum or plasma (37). One of the most common isolation methods is ultracentrifugation, which is a method of applying a high centrifugal force to a fluid to allow the deposition of particles according to their size. First, a low-speed centrifugation step is carried out to remove dead cells and other cell debris, while a high-speed step at increasing speeds (~10,000 x g) is required to deposit larger EVs as micro vesicles, followed by a high-speed ultracentrifugation step at 100,000 x g to pellet smaller EVs such as exosomes (38). However, this method is time-consuming (4-5 h), requires an ultracentrifuge and results in a comparatively poor recovery of EVs, ranging from 5-25% (39). Alternatively, exosomes are isolated from biological fluids by altering the solubility or dispersibility of the exosomes. For this purpose, water-excluding polymers, such as polyethylene glycol (PEG) are used. For the incubation of the sample, a precipitating solution with PEG and a molecular weight of 8000 Da is used. The precipitated exosomes are isolated by filtering or low-speed centrifugation following an overnight incubation period at 4˚C. Exosome precipitation is easy and does not require special equipment. It allows for the easy integration of this technique with clinical practice by using existing technology. The major drawback of exosome precipitation by polymers is the co-precipitation of other impurities such as proteins and polymeric materials (40).

It has been demonstrated that EVs may be effectively separated from contaminated plasma proteins and high-density lipoproteins (HDLs) using size exclusion chromatography (SEC) (41). SEC has a number of practical and technological drawbacks, although it is the preferred technique for separating relatively uncontaminated EVs from plasma. Only EVs larger than the pore size of the matrix, 70 nm for CL-2B Sepharose, can be effectively isolated with SEC. EV-rich fractions may still contain trace amounts of other lipoproteins, such as chylomicrons (100-600 nm) and very low-density lipoproteins (30-80 nm), even though they are devoid of HDLs. The relatively low yield of vesicles and the dilution of the purified sample, which necessitates additional steps and could result in the decline of the yield of the sample, are two more limitations of SEC procedure (41).

In general, various isolation methods have different challenges in terms of particle sizes, the concentration of the particles and purity of the isolated particles, etc. Therefore, obtaining pure exosomes fraction remains challenging. Researchers have to find a middle ground between specificity and yield in selecting an appropriate exosome isolation method (37). The short half-life of exosomes renders it difficult to both effectively deliver them to sites of injury and maintain their activity at the desired location (42).

## 7. Current challenges and limitations

Although the most common isolation methods, including ultracentrifugation, SEC and precipitation have been investigated, standardization will be necessary to improve the yield and purity of the exosomes. Ultracentrifugation is the standard method used for the isolation of exosomes; yet, it is a time-consuming process that requires expensive equipment (ultracentrifuge), apart from being associated with a lower exosome recovery rate. Viscous biological fluids may require extended periods of centrifugation and even multiple ultracentrifugation steps, which may damage the exosomes (38). Furthermore, the centrifugal force can readily mechanically damage exosomes, rendering it challenging to preserve their bioactivity and morphological integrity (43). SEC is most well suited for further studies due to high levels of purity and the conservation of exosome structure, although its low yields and dilution render it unsuitable for scaling up (41). On the other hand, precipitation techniques are simple, rapid and do not require any special equipment; thus. They are appropriate for routine and clinical applications; however, their major disadvantage is low specificity caused by the co-precipitation of non exosomal material (40).

The purification of exosomes with high purity, yield and recovery remains challenging owing to the heterogeneity of exosomes and the complexity of the matrix. The currently available methods for separating exosomes are mainly dependent on their size and surface-specific proteins. Although size-based isolation does not involve labelling, their purity is low. Specifically, the ensuing tests will be impacted by co-isolated non-exosomal functional vesicles. The purification and isolation of high-quality exosomes are currently being achieved using different methods, such as ultracentrifugation along with SEC. The entire approach performs exceptionally well in enhancing the label-free recovery of highly pure exosomes (43). To overcome these challenges, adherence to established guidelines, such as the Minimal Information for Studies of Extracellular Vesicles (MISEV) is strongly recommended (44).

## 8. Recent advancements in surface engineering of exosomes

The surface modifications of exosomes can be classified as genetic, chemical, or physical modifications. Genetic modifications comprise the genetic manipulation of donor cells to generate specific ligands or proteins to coat the exosome surfaces to achieve targeted delivery to the desired tissues. On the other hand, chemical modifications are based on the conjugation process via click or covalent modifications that allow the attachment of drugs or peptides to enhance therapeutic capabilities (35,36).

Physically driven modifications, such as electroporation and sonication increase the loading capacity and are commonly used for cargo enrichment. However, these modifications may lead to exosomal damage due to mechanical disruption and aggregation of the exosome particles. Non-covalent approaches, such as hydrophobic insertions and ligand-receptor binding allow for functionalization without compromising the integrity of the lipid bilayer (35).

Moreover, advancements in material science technologies have allowed for more sophisticated exosome modification strategies to enhance therapeutic functions. Specifically, the coating of exosomes using metal-polyphenol networks improves their stability, protects exosomal cargos and increases effectiveness (31).

Furthermore, hydrogel-mediated exosome delivery platforms have been developed, which offer controlled and localized release of exosomes, resulting in the improved localization of the exosomes to the target organ and enhanced regenerative capacity (18,30). Moreover, exosome mimetics prepared using extrusion techniques have also been explored due to their scalability and suitability for mass production (36).

## 9. Conclusion

The various functions of exosomes in osteogenesis, angiogenesis, immunomodulation and repair of soft tissues have positioned them as an emerging cell-free therapeutic treatment for periodontal regeneration. Preclinical and *in vitro* studies (5,20) have revealed that they possess the ability to regenerate periodontal tissues, with outcomes comparable to or even superior to those from conventional regenerative treatments. Exosomes possess special advantages over stem cell therapy, such as lower immunogenicity, easier storage and higher safety for use in the clinic. Nevertheless, there are several issues that restrict their use, including the lack of well-established protocols for isolation and characterization, unclear dosage optimization, and challenges to large-scale production and regulatory approval in routine clinical practice. These limitations may be overcome and their therapeutic effectiveness enhanced through the assistance of advancements in bioengineering, exosome manipulation and biomaterial-mediated delivery systems. In periodontal regenerative treatment, exosomes are a novel and highly effective tool as a whole; however, further well-designed clinical trials are warranted to validate their safety, efficacy and long-term outcomes.

## Figures and Tables

**Table I:** Summary of studies on the role of exosomes in periodontal tissue regeneration.

Authors	Year of publication	Aim/objective	Type of study	Mode/system	Key findings/results	(Refs.)
Zhang *et al*	2021	Compare 3D vs. 2D MSC exosome effects on periodontal healing	Experimental	Rat periodontitis; 3D MSC culture	3D culture boosted exosome yield and regenerative efficacy	(13)
Sun *et al*	2022	Test gingival MSC exosomes on inflammation & repair	Experimental	LPS-stimulated PDLSCs; rat periodontitis	Suppressed IL-6/TNF-α; Improved periodontal regeneration	(26)
Zhao *et al*	2022	Evaluate PDLSC-Exos in Gel-Alg hydrogel for bone repair	Experimental	Rat alveolar defect; BMSC culture	Hydrogel-Exos improved bone fill and osteogenesis	(27)
Lin *et al*	2022	Summarize exosome-base d therapies	Systematic review	Preclinical/early clinical	Strong preclinical support; need human trials	(10)
Huang *et al*	2023	Study inflammatory preconditioning on exosome function	Experimental	LPS-periodontitis model	TNF-α priming enhanced M2 polarization; prevented bone loss	(21)
Mousavi *et al*	2023	Review MSC-exosome roles across tissues	Review	Multisystem	Highlighted paracrine signalling; translational potential	(28)
Albougha *et al*	2024	Test osteogenic ability of PDLSC-exos	Experimental	Osteoblast-like cells; rat calvarial defect	Stimulated mineralization and defect healing	(29)
Peng *et al*	2020	Review exosome effects on relapse and remodelling	Review	Bone remodelling models	Balanced RANKL/OPG axis; promoted bone regeneration	(15)
Wang *et al* (PMC)	2024	Review composite scaffolds with exosomes	Review	Biomaterial studies	Scaffold-exosome synergy - improved retention and osteogenesis	(30)
Deng *et al*	2024	To outline the role of exosomes and exosome loaded scaffolds in bone regeneration	Review	Based on recent *in vitro*, *in vivo* and preclinical studies	Exosome enhance osteogenesis, angiogenesis, and immune modulation	(18)
Zhou *et al*	2025	Create metal-polyphenol-network exosome delivery system	Experimental	PDLSC + rat defect	Prolonged release-superior periodontal regeneration	(31)
Chen *et al*	2025	Examine how LPS preconditioning alters exosomal miRNA cargo	Experimental	PDLSC/macrophage + rat periodontitis	miR-21/miR-146a-M2 polarization and bone healing	(32)

MSC, mesenchymal stem cell; LPS, lipopolysaccharide; PDLSCs, periodontal ligament stem cells.

## Data Availability

Not applicable.

## References

[1] Koca-Ünsal RB, Chaurasia A (2022). Roles of exosomes in regenerative periodontology: A narrative review. Mol Biol Rep.

[2] Elsayed R, Elashiry M, Liu Y, El-Awady A, Hamrick M, Cutler CW (2021). Porphyromonas gingivalis provokes exosome secretion and paracrine immune senescence in bystander dendritic cells. Front Cell Infect Microbiol.

[3] Chew JRJ, Chuah SJ, Teo KYW, Zhang S, Lai RC, Fu JH, Lim LP, Lim SK, Toh WS (2019). Mesenchymal stem cell exosomes enhance periodontal ligament cell functions and promote periodontal regeneration. Acta Biomater.

[4] Wang X, Chen J, Tian W (2022). Strategies of cell and cell-free therapies for periodontal regeneration: The state of the art. Stem Cell Res Ther.

[5] Chen L, Zhu S, Guo S, Tian W (2023). Mechanisms and clinical application potential of mesenchymal stem cells-derived extracellular vesicles in periodontal regeneration. Stem Cell Res Ther.

[6] Jing L, Wang HY, Zhang N, Zhang WJ, Chen Y, Deng DK, Li X, Chen FM, He XT (2025). Critical roles of extracellular vesicles in periodontal disease and regeneration. Stem Cells Transl Med.

[7] Lei F, Li M, Lin T, Zhou H, Wang F, Su X (2022). Treatment of inflammatory bone loss in periodontitis by stem cell-derived exosomes. Acta Biomater.

[8] Wang X, He L, Huang X, Zhang S, Cao W, Che F, Zhu Y, Dai J (2021). Recent progress of exosomes in multiple myeloma: Pathogenesis, diagnosis, prognosis and therapeutic strategies. Cancers (Basel).

[9] Kong Q, Wang Y, Jiang N, Wang Y, Wang R, Hu X, Mao J, Shi X (2024). Exosomes as promising therapeutic tools for regenerative endodontic therapy. Biomolecules.

[10] Lin H, Chen H, Zhao X, Ding T, Wang Y, Chen Z, Tian Y, Zhang P, Shen Y (2022). Advances of exosomes in periodontitis treatment. J Transl Med.

[11] Gurung S, Perocheau D, Touramanidou L, Baruteau J (2021). The exosome journey: From biogenesis to uptake and intracellular signalling. Cell Commun Signal.

[12] Zhang Y, Liu Y, Liu H, Tang WH (2019). Exosomes: Biogenesis, biologic function and clinical potential. Cell Biosci.

[13] Zhang Y, Chen J, Fu H, Kuang S, He F, Zhang M, Shen Z, Qin W, Lin Z, Huang S (2021). Exosomes derived from 3D-cultured MSCs improve therapeutic effects in periodontitis and experimental colitis and restore the Th17 cell/Treg balance in inflamed periodontium. Int J Oral Sci.

[14] Novello S, Pellen-Mussi P, Jeanne S (2021). Mesenchymal stem cell-derived small extracellular vesicles as cell-free therapy: Perspectives in periodontal regeneration. J Periodontal Res.

[15] Peng Q, Yang JY, Zhou G (2020). Emerging functions and clinical applications of exosomes in human oral diseases. Cell Biosci.

[16] Liang B, Liang JM, Ding JN, Xu J, Xu JG, Chai YM (2019). Dimethyloxaloylglycine-stimulated human bone marrow mesenchymal stem cell-derived exosomes enhance bone regeneration through angiogenesis by targeting the AKT/mTOR pathway. Stem Cell Res Ther.

[17] Liu C, Xiao K, Xie L (2022). Advances in the use of exosomes for the treatment of ALI/ARDS. Front Immunol.

[18] Deng L, Liu Y, Wu Q, Lai S, Yang Q, Mu Y, Dong M (2024). Exosomes to exosome-functionalized scaffolds: A novel approach to stimulate bone regeneration. Stem Cell Res Ther.

[19] Nakao Y, Fukuda T, Zhang Q, Sanui T, Shinjo T, Kou X, Chen C, Liu D, Watanabe Y, Hayashi C (2021). Exosomes from TNF-α-treated human gingiva-derived MSCs enhance M2 macrophage polarization and inhibit periodontal bone loss. Acta Biomater.

[20] Chen M, Huang B, Su X (2025). Mesenchymal stem cell-derived extracellular vesicles in periodontal bone repair. J Mol Med (Berl).

[21] Huang X, Li Y, Liao H, Luo X, Zhao Y, Huang Y, Zhou Z, Xiang Q (2023). Research advances on stem cell-derived extracellular vesicles promoting the reconstruction of alveolar bone through RANKL/RANK/OPG pathway. J Funct Biomater.

[22] Bian X, Ma K, Zhang C, Fu X (2019). Therapeutic angiogenesis using stem cell-derived extracellular vesicles: An emerging approach for treatment of ischemic diseases. Stem Cell Res Ther.

[23] Hellström M, Kalén M, Lindahl P, Abramsson A, Betsholtz C (1999). Role of PDGF-B and PDGFR-beta in recruitment of vascular smooth muscle cells and pericytes during embryonic blood vessel formation in the mouse. Development.

[24] Ahmad P, Estrin N, Farshidfar N, Zhang Y, Miron RJ (2025). Mechanistic insights into periodontal ligament stem cell-derived exosomes in tissue regeneration. Clin Oral Investig.

[25] Kang M, Huang CC, Lu Y, Shirazi S, Gajendrareddy P, Ravindran S, Cooper LF (2020). Bone regeneration is mediated by macrophage extracellular vesicles. Bone.

[26] Sun J, Wang Z, Liu P, Hu Y, Li T, Yang J, Gao P, Xu Q (2022). Exosomes derived from human gingival mesenchymal stem cells attenuate the inflammatory response in periodontal ligament stem cells. Front Chem.

[27] Zhao Y, Gong Y, Liu X, He J, Zheng B, Liu Y (2022). The experimental study of periodontal ligament stem cells derived exosomes with hydrogel accelerating bone regeneration on alveolar bone defect. Pharmaceutics.

[28] Mousavi E, Khosravi A, Sedigh SS, Mayanei SAT, Banakar M, Karimzadeh M, Fathi A (2023). Exosomes derived from mesenchymal stem cells: Heralding a new treatment for periodontitis?. Tissue Cell.

[29] Albougha MS, Sugii H, Adachi O, Mardini B, Soeno S, Hamano S, Hasegawa D, Yoshida S, Itoyama T, Obata J, Maeda H (2024). Exosomes from human periodontal ligament stem cells promote differentiation of osteoblast-like cells and bone healing in rat calvarial bone. Biomolecules.

[30] Wang T, Zhou Y, Zhang W, Xue Y, Xiao Z, Zhou Y, Peng X (2024). Exosomes and exosome composite scaffolds in periodontal tissue engineering. Front Bioeng Biotechnol.

[31] Zhou ZY, Li ZB, Shi NS, Feng SS, Han YR, Liu MT, Chen H, Li JH, Ge SH, Yu Y (2026). Metal-polyphenol network-engineered mesenchymal stem cell-derived exosome mimetics mediate inflammatory/immune regulation for enhanced periodontal tissue regeneration. Biomaterials.

[32] Chen L, Zhang J, Yu J, Guo S, Tian W (2025). LPS pretreated dental follicle stem cell derived exosomes promote periodontal tissue regeneration via miR-184 and PPARα-Akt-JNK signaling pathway. Stem Cell Res Ther.

[33] Yue C, Cao J, Wong A, Kim JH, Alam S, Luong G, Talegaonkar S, Schwartz Z, Boyan BD, Giannobile WV (2022). Human bone marrow stromal cell exosomes ameliorate periodontitis. J Dent Res.

[34] Shen Z, Kuang S, Zhang Y, Yang M, Qin W, Shi X, Lin Z (2020). Chitosan hydrogel incorporated with dental pulp stem cell-derived exosomes alleviates periodontitis in mice via a macrophage-dependent mechanism. Bioact Mater.

[35] Chao T, Zhao J, Gao R, Wang H, Guo J, Gao Z, Wang Y (2025). Exosome surface modification and functionalization: A narrative review of emerging technologies and their application potential in precision medicine. Adv Technol Neurosci.

[36] Zeng H, Guo S, Ren X, Wu Z, Liu S, Yao X (2023). Current strategies for exosome cargo loading and targeting delivery. Cells.

[37] Skuratovskaia D, Vulf M, Khaziakhmatova O, Malashchenko V, Komar A, Shunkin E, Gazatova N, Litvinova L (2021). Exosome limitations in the treatment of inflammatory diseases. Curr Pharm Des.

[38] Martins TS, Vaz M, Henriques AG (2023). A review on comparative studies addressing exosome isolation methods from body fluids. Anal Bioanal Chem.

[39] Momen-Heravi F, Balaj L, Alian S, Mantel PY, Halleck AE, Trachtenberg AJ, Soria CE, Oquin S, Bonebreak CM, Saracoglu E (2013). Current methods for the isolation of extracellular vesicles. Biol Chem.

[40] Li P, Kaslan M, Lee SH, Yao J, Gao Z (2017). Progress in exosome isolation techniques. Theranostics.

[41] Stranska R, Gysbrechts L, Wouters J, Vermeersch P, Bloch K, Dierickx D, Andrei G, Snoeck R (2018). Comparison of membrane affinity-based method with size-exclusion chromatography for isolation of exosome-like vesicles from human plasma. J Transl Med.

[42] Tzng E, Bayardo N, Yang PC (2023). Current challenges surrounding exosome treatments. Extracell Vesicle.

[43] Gao J, Li A, Hu J, Feng L, Liu L, Shen Z (2023). Recent developments in isolating methods for exosomes. Front. Bioeng. Biotechnol.

[44] Théry C, Witwer KW, Aikawa E, Alcaraz MJ, Anderson JD, Andriantsitohaina R, Antoniou A, Arab T, Archer F, Atkin-Smith GK (2018). Minimal information for studies of extracellular vesicles 2018 (MISEV2018): A position statement of the International Society for Extracellular Vesicles and update of the MISEV2014 guidelines. J Extracell Vesicles.

